# The folded, bipaddled pectoralis major myocutaneous flap for complex oral cavity defects: Undiminished relevance in the era of free flaps

**DOI:** 10.1016/j.jpra.2020.11.007

**Published:** 2020-11-25

**Authors:** Vidya Konduru, Amit Jiwan Tirkey, Kingston Samy, Kiran Kumar Devarakonda, Rajinikanth Janakiraman

**Affiliations:** a Department of Head and Neck Surgery, Unit-2, Christian Medical College, Vellore, Tamil Nadu, 632004 India; b Christian Medical College, Vellore, Tamil Nadu, 632004 India

**Keywords:** Bipaddled pectoralis major myocutaneous flap, Head and neck reconstruction, Pedicled flaps, Head and neck cancer, Folded pectoralis major myocutaneous flap

## Abstract

Oral cancer often presents at an advanced stage, requiring extensive resection and complex reconstruction, such as free tissue transfers, which may not be available in a remote or resource-constrained facility. The common alternative in these cases is the use of the workhorse flap, the pectoralis major myocutaneous (PMMC) flap for lining and a second regional flap for cover. The results are variable, increase operative time and cost, and may cause additional donor site morbidity.

We present a series of patients who underwent reconstruction for complex oral cavity and neck defects with a single PMMC flap with a unique design, folded or bipaddled to serve as both lining and cover. Pre- and post-operative data pertaining to patients with oral cancer who were selected to undergo bipaddled PMMC flap reconstruction in our unit between January 2017 and July 2019 were collected and analysed.

Of the 41 patients, 28 were males and 13 were females. The surgical resection involved full-thickness excision of primary tumour and involved skin (face or neck) for oral cancers. The size of skin paddle harvested ranged from 8 to 15 cm horizontally to 6 to 22 cm vertically. Usually, the distal part of the skin paddle formed the mucosal lining and the proximal formed the skin cover. Complication rates in the immediate postoperative period and on initial follow-up visits were comparable to a conventional PMMC flap.

Reconstruction of complex head and neck defects requiring mucosal lining and skin cover can be achieved with a single stage, bipaddled PMMC flap, a reliable and easily learnt alternative to technically demanding free tissue transfers. The complication rate observed in our series is remarkably low, even in females. With a proper design of the flap and appropriate orientation of the skin paddle, excellent results can be achieved with a bipaddled PMMC flap.

## Introduction

Cancer of the oral cavity continues to be a devastating disease in the Indian subcontinent. Locally advanced oral cancers require multimodality treatment and choosing the most appropriate reconstructive procedure is of the utmost importance for rehabilitation. The reconstruction of complex, full-thickness defects of the head and neck region following extirpation of oral cancer is a challenge in balancing form and function. The ideal method of reconstruction by free tissue transfer may be elusive to many centres across the developing world. After the description of its use for head and neck reconstruction by Ariyan in 1979,^1^ the “workhorse” flap of head and neck reconstruction – the pectoralis major myocutaneous (PMMC) flap – remains a suitable alternative to the more expensive, time-consuming and technically demanding free tissue transfer. Moreover, it is a valuable tool for salvage following failure of free flaps.^2^^,^^3^

The skin paddle of the PMMC flap was originally used to form the lining of the oral cavity defect, along with another pedicled flap, most often the deltopectoral flap, to form the skin cover for a through-and-through defect. Ariyan had also designed two skin paddles to be used for intraoral lining and extraoral cover for full-thickness defects of the cheek.^4^ The flap can also be folded or bipaddled to achieve the same purpose. Use of a single flap to serve both functions reduces operative time, cost and donor site morbidity. Various designs of the bipaddled PMMC skin paddle have been described in literature, which enable safe harvest and improved reliability, even when large skin paddles are harvested.^5^, ^6^, ^7^, ^8^ We have developed our own modifications in flap design to enhance the outcomes at donor and recipient sites. We present our experience with the use of the bipaddled PMMC flap for reconstruction of complex oral cavity and neck defects.

## Materials and methods

A total of 41 consecutive patients with oral cavity cancers, operated between January 2017 and July 2019, were included in this study. These patients had tumour infiltrating the skin of the face or neck, from the primary tumour or a nodal mass, either clinically suspicious (puckering, dimpling) or obvious (ulceration, fungation, orocutaneous fistula). They were selected to undergo reconstruction with the bipaddled PMMC flap because they were either unfit for a free tissue transfer, or had financial constraints. Data pertaining to patient demographics, diagnosis and stage of the disease and dimensions of the defect following resection was collected and analysed. Patients were followed-up after surgery for post-operative complications at the donor and recipient sites, and any delay in the initiation of adjuvant radiation therapy due to flap complications was noted. Only descriptive statistics were employed for analysis. The study was approved by the Institutional Research Board (IRB) and Ethics Committee. We have followed the guidelines described in the Helsinki Declaration and did not use any animal subjects in this study.

### Operative technique

**Fig. 1:**
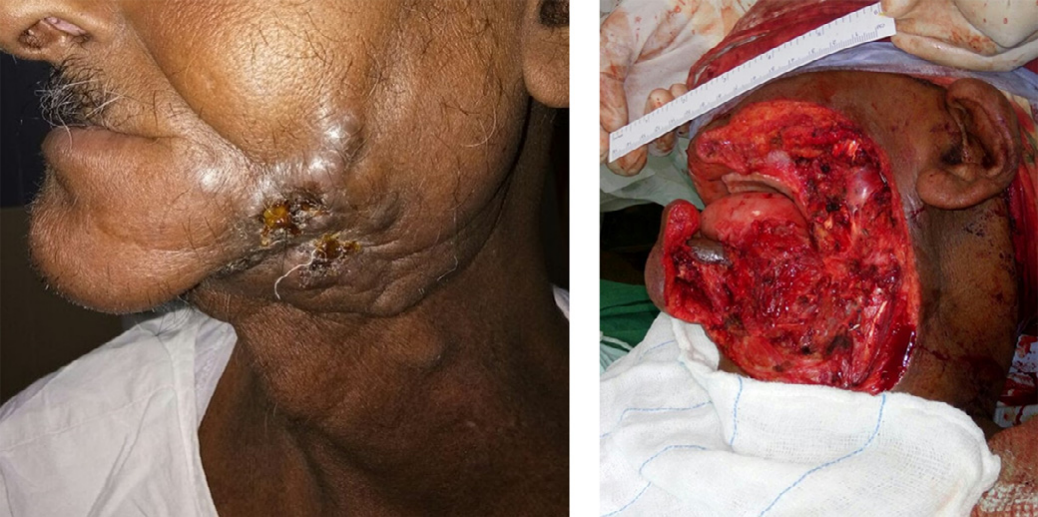
Pre-operative photograph of the lesion in a patient with recurrent carcinoma left buccal mucosa planned for salvage surgery (A). Intraoperative photograph post-resection (B), demonstrating the challenges of reconstructing an extensive and composite defect.

The gingivobuccal complex (GBC), which includes buccal mucosa, upper and lower gingivobuccal sulci, lower alveolus and retromolar trigone, is the most common subsite for oral cancer among patients presenting to our institution. Resection for such tumours includes wide local excision of the tumour and the involved skin and segmental- or hemimandibulectomy, depending upon the location of the tumour. Part of the upper alveolus, oral commissure and lips are resected when necessary, for adequate surgical margins. When the skin of the neck is involved, it is resected by incorporating it into the incision for neck dissection. The resultant defects of the skin and mucosa are measured in two dimensions and the flap designed accordingly (Fig. 1).

**Fig. 2:**
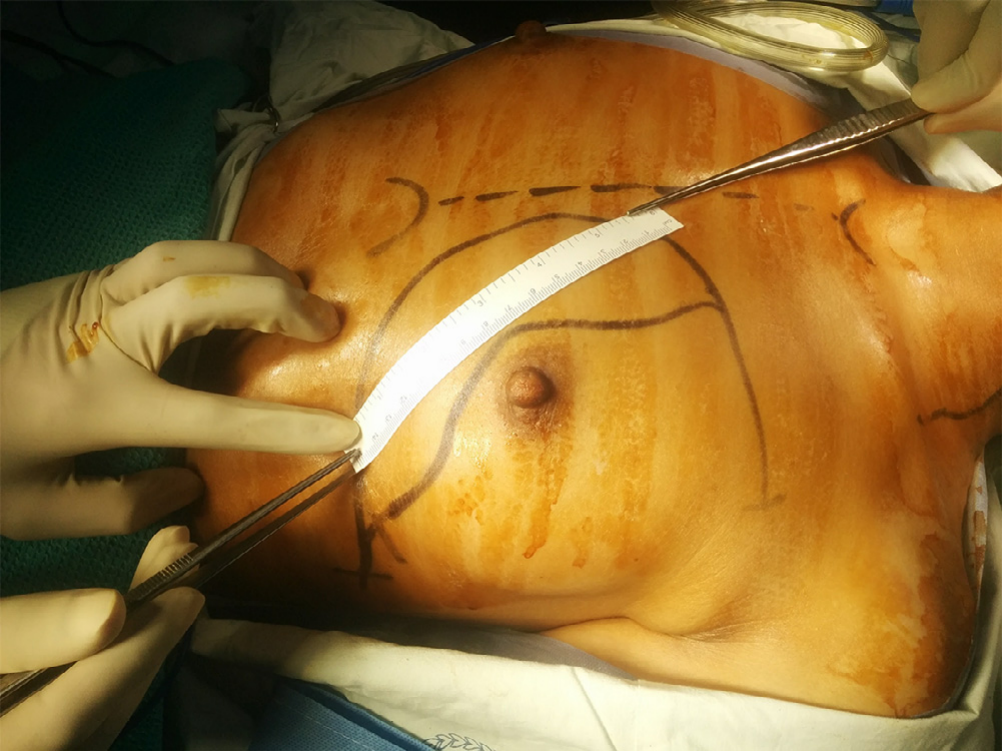
Skin paddle designed like a “boomerang” or “banana” within a laterally facing “C” marked on the pectoral region.

**Fig. 3:**
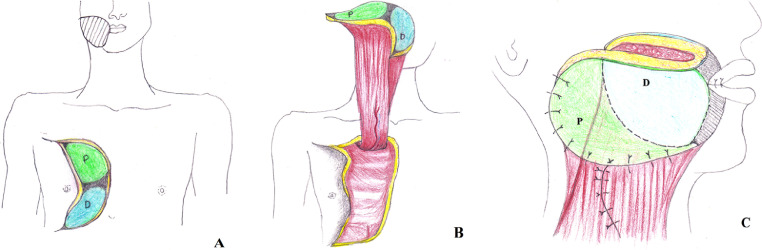
Illustration showing the flap design (A) and orientation of flap for inset (B and C). Distal skin paddle (D) coloured in green and proximal skin paddle (P) in blue; areas de-epithelialized before inset coloured grey with cross-hatching.

**Fig. 4:**
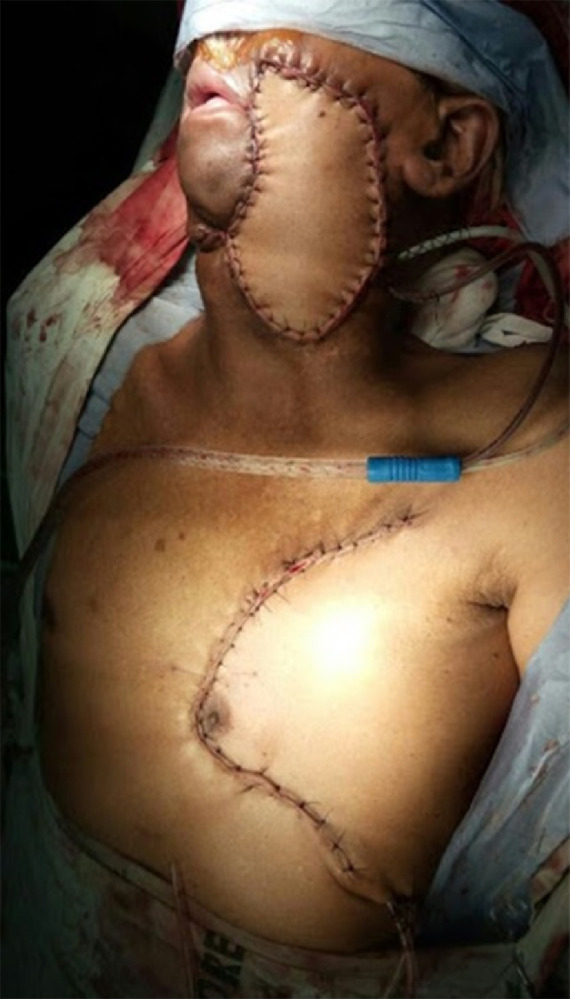
Immediate post-operative photograph of the same patient shown in Fig. 2. Closure of large defects at the donor site is possible without the need for skin grafts, with the “boomerang”-shaped skin paddle design.

**Fig. 5:**
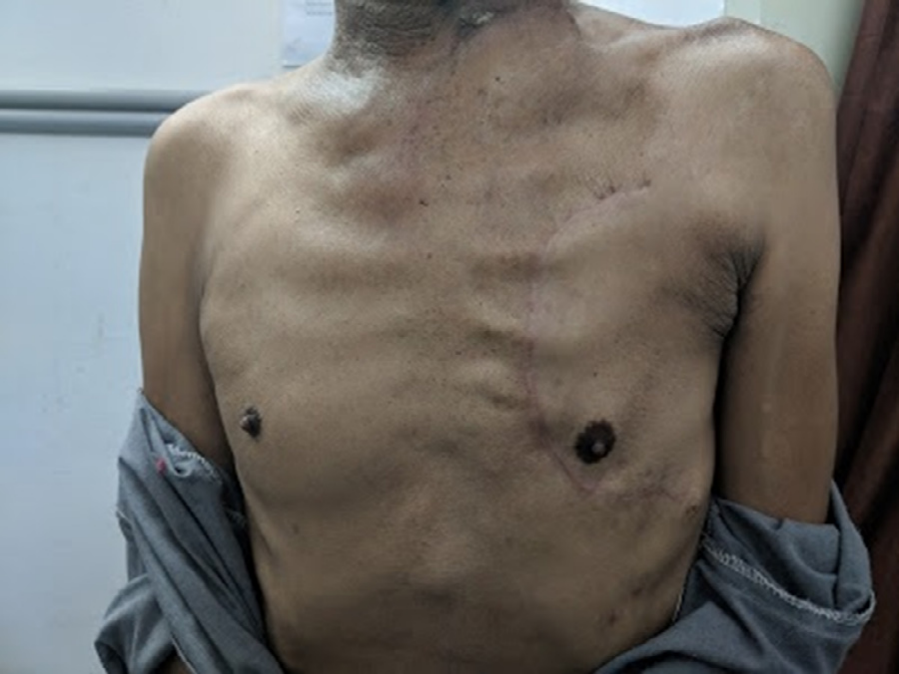
Sacrifice of the nipple-areolar complex is avoided during flap harvest and the “C”-shaped donor-site closure allows for the symmetry of the chest, as seen in this patient 6 months after surgery.

The skin paddle of the flap in each patient was designed like a “boomerang” or “banana” within a laterally facing “C” marked on the pectoral region, as demonstrated in Fig. 2. The skin paddle was centred inferomedial to the nipple-areolar complex (NAC), with extensions inferolaterally and medially as required. The proximal and distal ends of the skin paddle were marked by the distance between the pivot point situated at the middle of the lower border of the clavicle and the most anterior and posterior points of the surgical defect respectively. The proximal and distal portions of the flap, assigned to form the skin cover and the mucosal lining, were measured to fit the respective defects, and the skin paddle drawn, as shown in Fig. 3. An additional 1–2 cm was included between the proximal and distal parts, to allow for the folding of the flap and to provide a surface on which to attach the upper and/or lower lips where the oral commissure required reconstruction. Large skin paddles could be harvested without incorporating the NAC, to avoid cosmetic disfigurement and psychological distress (Fig. 4). Placing the skin paddle within the “C” ensured that the donor site could be closed primarily without craniocaudal displacement of the NAC (Fig. 5). The incision preserved the skin paddle and perforators to the deltopectoral flap, the salvage option. The “C” can be extended laterally to widely undermine and approximate the wound edges to each other, enabling the harvest of very wide flaps without requiring skin grafts to close the donor site defects (Fig. 4). Tapering the ends of the skin paddle adheres to the basic principles of plastic surgery and prevents dog ears upon closing the donor site.

**Fig. 6:**
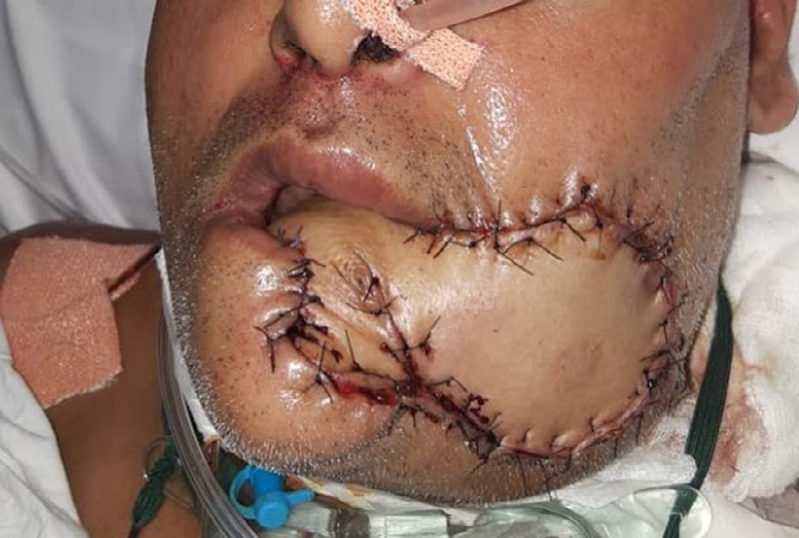
Bipaddled osteomyocutaneous flap used for reconstruction of an anterior segmental mandibulectomy defect.

**Fig. 7:**
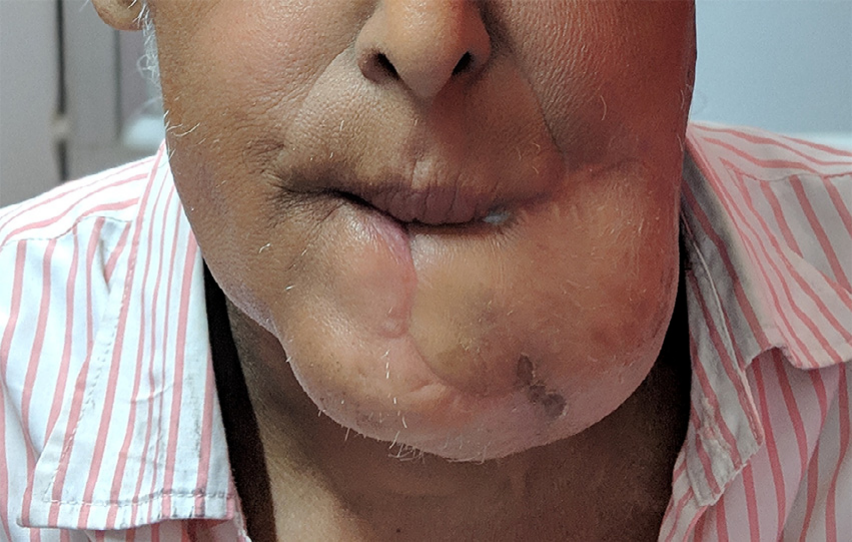
Long-term outcome, the same patient shown in Fig. 5, at one year of follow-up after completion of adjuvant radiation, showing good oral competence and jaw contour.

**Fig. 8:**
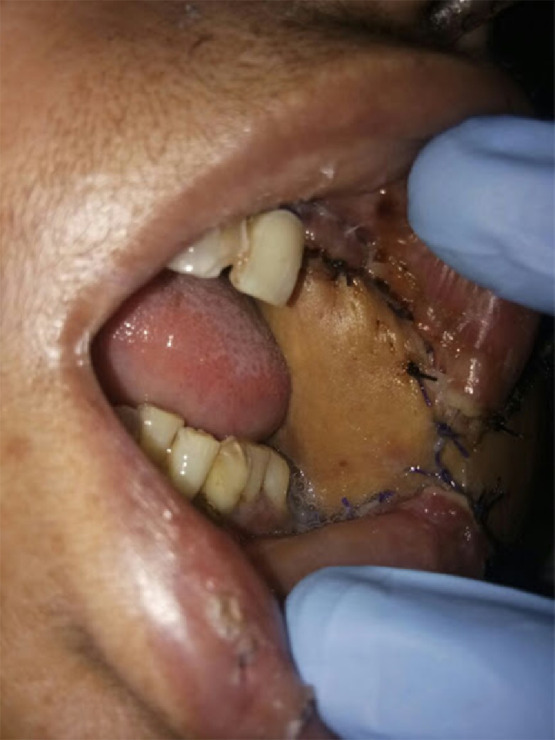
Inset of upper and lower lips onto de-epithelialized portions of the flap, to recreate the oral commissure. Overcorrection of the neocommissure ensures symmetry of lips post-operatively, despite sagging of the flap.

**Fig. 9:**
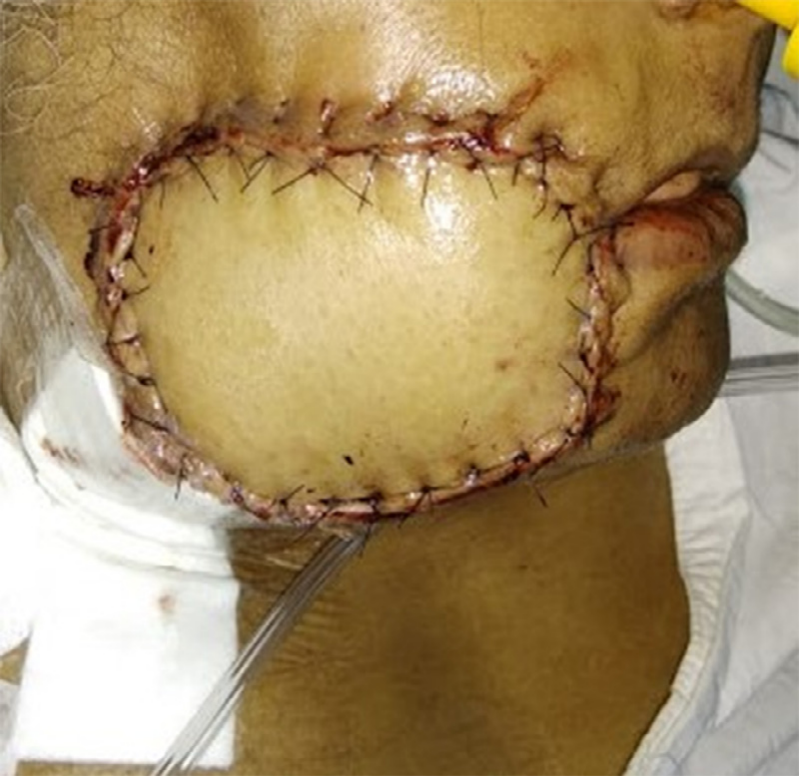
Tension-free inset of a large bipaddled flap in a female patient minimizes the risk of congestion and flap loss. Bulk of the folded flap can counteract the mandibular deviation following hemimandibulectomy.

Flap harvest proceeded similar to the original technique described by Ariyan and energy devices (harmonic scalpel, bipolar scissors and Ligasure) were employed to minimize operative time. When the distal portion of the flap overlay the anterior rectus sheath, or the fascia over the external oblique and serratus anterior, attempts were made to minimize this portion to less than a third of the skin paddle. For harvest of an osteomyocutaneous flap, the sixth rib was harvested (Figs. 6, 7). The ipsilateral sternocleidomastoid muscle was removed to accommodate the pedicle in all cases. The distal portion of the flap formed the intraoral lining and the proximal portion formed the extra-oral cover. The flap was inset starting at the most posterior point of the defect. Tapered triangular ends of the skin paddle were trimmed at this point, to avoid necrosis. The inset continued by placing sutures alternately on the superior and inferior edges, until the oral commissure was reached. If the oral commissure was sacrificed, a new commissure was created by insetting the cut ends of the upper and lower lips on to matching semi-circular de-epithelialized areas on the flap (Fig. 8). Where the oral commissure was not resected, a de-epithelialized strip between the intra- and extra-oral parts of the flap facilitated folding of the flap without discontinuity of the skin paddle. Typically, the commissure was overcorrected to avoid long-term oral incompetence when the flap becomes slack (Fig. 8). The inset was completed with sutures placed alternately on either sides of the extra-oral skin paddle, which enabled tension-free inset, without forcing the paddle to conform to the defect by placing stay sutures (Fig. 9).

## Results

Of the 41 patients included in the study, 28 (68%) were males and 13 (32%) were females. Age of the patients ranged from 19 to 69 years. All patients suffered from squamous cell carcinoma (SCC) of various oral cavity subsites, except one 19-year-old female, who was diagnosed with synovial sarcoma of the mandible. One patient had SCC of the tongue and 39 had SCC of the GBC. Skin involvement was either suspected clinically (44%) or was obvious at the time of surgery (56%). All the patients had clinical stage IV disease, as per the 8th edition AJCC TNM staging, due to the involvement of skin, bone (T4) or extranodal extension from cervical nodes to the overlying skin (N3b, *n* = 3).

In patients with GBC cancers, the resection comprised of a wide local excision including the involved skin and segmental- or hemimandibulectomy, depending upon the location of the tumour. Part of the upper alveolus, oral commissure and lips were resected when necessary for adequate surgical margins. When the skin of the neck was involved (*n* = 3), it was resected separately, by incorporating it into the incision for neck dissection. The resultant defects of skin and mucosa were measured in two dimensions and the flap designed accordingly.

The maximum mucosal defect encountered was 8 × 6 cm, and the maximum skin defect was 16 × 10 cm. The maximum size of the skin paddle harvested was 22 × 15 cm (average 13.1 × 6.7 cm). Donor site was closed primarily in all cases. Two patients with anterior segmental mandibulectomy had reconstruction with a rib-PMMC flap. Resection and reconstruction, when done as a two-team approach, resulted in operative times being reduced from 6 to 7 h to 3.5–4 h.

As we gained experience, we learnt that the most natural orientation of the flap was to inset the distal portion to form the intraoral lining and the proximal portion to form the extra-oral cover (Fig. 3). In 10 cases, however, it was possible to rotate the pedicle to place the proximal part of the flap intraorally. This was not always reliable, and the pedicle became taut when forced to conform, and resulted in partial flap loss in two cases.

Table 1.

**Table 1:** Demographic and intraoperative data of the 41 cases of bipaddled pectoralis major myocutaneous flap reconstruction.

Number of patients (*n* = 41)	
**Age**	
Range	19–69 years
Average age in males	48.4 years
Average age in females	48.3 years
**Gender**	
Males	28
Females	13
**Diagnosis**	
SCC Buccal mucosa	24
SCC lower alveolus	6
SCC lower gingivobuccal sulcus	9
Synovial sarcoma of mandible	1
SCC tongue	1
**Laterality**	
Right	18
Left	23
**Clinical skin involvement**	
Absent	6
Doubtful	12
Present	23
**Pathological skin involvement**	
No tumour in specimen	1
Involved	19
Not involved (0.5 to 1.7 cm away)	21
**Type of mandibulectomy**	
Anterior segmental	2
Posterior segmental	3
Hemimandibulectomy	20
Hemimandibulectomy, not crossing midline	13
Extended hemimandibulectomy	2
Reverse marginal mandibulectomy	1
**Inferior partial maxillectomy**	
Yes	19
No	22
**Infratemporal fossa clearance**	
Yes	5
No	36
**Mucosal defect (in cm.)**	
Maximum	8 x 7
Minimum	4 x 3
Average	5.46 x 4.4
**Skin defect (in cm.)**	
Maximum	16 x 10
Minimum	3 x 2
Average	4.8 x 4.5
**Oral commissure resected**	
Yes	19
No	22
**Flap size (in cm.)**	
Maximum	22 x 15
Minimum	8 x 6
Average	13.1 x 6.7
**Rib harvested**	
Yes	2
No	39
**Flap orientation at inset**	
Distal end intra-oral	31
Proximal end intra-oral	10

Flap complications occurred mostly at the recipient site (Table 2). Intra-oral wound dehiscence was noted in three patients, and partial loss of intra- and extra-oral components was noted in three patients each. None of the patients had total flap loss. Minor complications like skin edge necrosis were managed conservatively. Five patients had a delay in the initiation of adjuvant therapy due to flap complications. When compared to single-paddle PMMC reconstructions performed in our unit, the complication rates for a bipaddled flap were found to be similar.

**Table 2:** Complications noted in the 41 cases of bipaddled pectoralis major myocutaneous flap reconstruction.

None	28
Intraoral wound dehiscence	3
Neck wound dehiscence	2
Loss of outer skin paddle	3
Loss of inner skin paddle	3
Flap edge necrosis	2
Total flap loss	0
Debridement required	
Yes	7
No	34
Secondary procedure required (secondary suturing, skin graft, second flap)	
Yes	6
No	35
Delay in adjuvant therapy due to wound complications (*n* = 35, planned for RT)	
Yes	5
No	30
Oral competence on follow-up	
Microstomia	1
Oral incompetence	4

## Discussion

Since the description of PMMC flap for head and neck reconstruction by Ariyan in 1979,^1^ its reliability was proven consistently in a number of studies.^9^^,^^10^^,^^11^ Although the ideal method of reconstruction for all kinds of head and neck surgical defects is free tissue transfer,^2^ the PMMC still finds utility across the world as a reconstructive option in both primary and salvage settings. The value of the flap in head and neck units in developing countries with low resources and high turn-over rates cannot be underestimated. Good vascularity, short learning curve and decreased need for specialized equipment are other notable advantages.^12^ Disadvantages include complications such as wound dehiscence, donor site morbidity and inferior recipient site outcomes compared to free tissue transfer techniques.^13^^,^^14^ Numerous modifications in design and technique have been proposed to adapt this versatile flap to the reconstructive needs of the patient and to minimize complications. The bipaddled PMMC is one such modification, which has been effectively utilized for complex, full-thickness defects in head and neck surgery, especially after extirpation of oral cancers.^3^^,^^9^^,^^10^

**Fig. 10:**
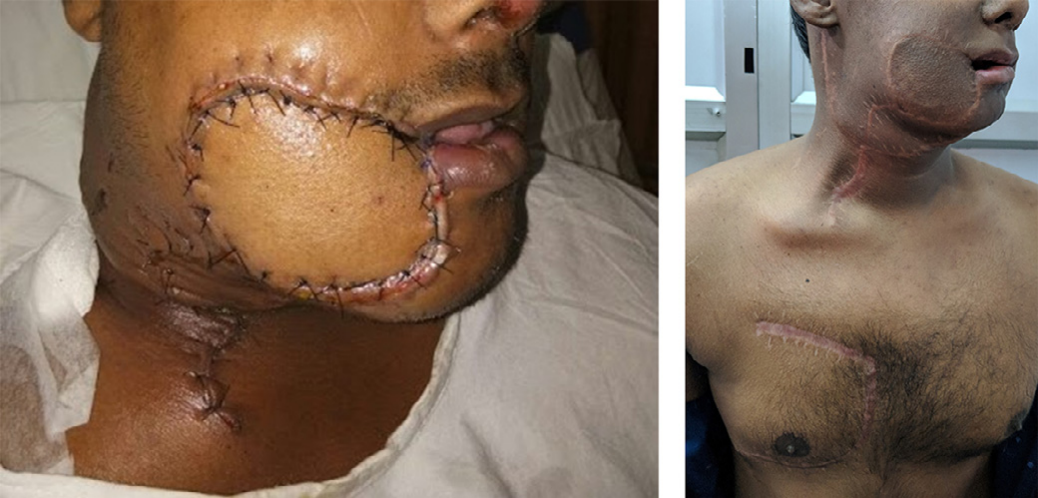
Immediate post-operative result (A) and long-term result after 11 months of completion of adjuvant therapy (B).

In this series on the bipaddled PMMC flap, we present our modification of the design, which aims to minimize donor site morbidity and maximize the area of skin paddle harvested. The design allows for placement of the skin paddle infero-medial to the nipple and areola, which has been reported to be more reliable in the literature.^15^ It also allows for the preservation of the nipple and areola, unlike most other studies, which, in our experience, makes a considerable difference in terms of cosmesis and psychological impact of the procedure on the patient. Tapering the ends of the skin paddle enables closure without dog ears. Primary closure of the donor site was possible in all our patients, due to the unique “boomerang”- or “banana”-shaped design of the skin paddle. De-epithelialization of the intervening skin between the extra- and intra-oral parts of the skin paddle was employed in every patient, without the need to disconnect the two paddles. When the oral commissure was sacrificed, the upper and/or lower lips were inserted on to the flap on semi-circular de-epithelialized areas matching the thickness of the lip, a modification of the technique described by Pai et al.^16^ At this juncture, care was taken to overcorrect the commissure to compensate for the lack of bony support and to account for post-operative drooping and resultant oral incompetence due to the bulk of the flap. Deviation of the jaw following hemimandibulectomy was partly compensated for by the bulk of the bipaddled flap^17^; especially for the female patients in our series (Fig. 9). Apart from troublesome intra-oral hair growth in males and the occasional lack of colour match, patients found the flap acceptable and achieved reasonable functional outcomes in terms of oral competence, speech and swallowing (Fig. 10).

Complications noted in our series were on par with single-paddle PMMC flaps performed by our unit. Females constitute 30% of our subjects, and large flaps were safely harvested and inset without much technical difficulty, even in patients with bulky breasts. As a unit, we have overcome the widely held fear of unreliability and complications in females undergoing a PMMC flap^18^ with our experience in successfully folding the PMMC.

## Conclusions

Reconstruction of complex head and neck and oral cavity defects can be expensive and time-consuming. Use of a bipaddled PMMC flap design can be as reliable an alternative as other complex forms of reconstruction, at a lower cost and, potentially lower or comparable rates of complications, as observed in our series. Better outcomes in terms of donor site closure, symmetry, avoidance of redundant skin and dog ears after closure were observed with our modified flap design. With careful assessment of defect size, our modified design for flap harvest, and appropriate orientation of the flap, excellent results can be achieved through a bipaddled PMMC flap.

## Funding sources

None.

## Ethical approval

This study was approved by the Institutional Research Board (IRB) and Ethics Committee.

## Declaration of Competing Interest

The authors declare that they have no known competing financial interests or personal relationships that could have appeared to influence the work reported in this paper.
